# The Automatic Context Measurement Tool (ACMT) to Compile Participant-Specific Built and Social Environment Measures for Health Research: Development and Usability Study

**DOI:** 10.2196/56510

**Published:** 2024-10-04

**Authors:** Weipeng Zhou, Amy Youngbloom, Xinyang Ren, Brian E Saelens, Sean D Mooney, Stephen J Mooney

**Affiliations:** 1 Department of Biomedical Informatics and Medical Education University of Washington Seattle, WA United States; 2 Department of Epidemiology Hans Rosling Center for Population Health University of Washington Seattle, WA United States; 3 Seattle Children's Research Institute Seattle, WA United States; 4 Center for Information Technology National Institutes of Health Bethesda, MD United States

**Keywords:** built environment, social environment, geocoding, GIS, geographic information systems, ACMT, automatic context measurement tool, linkage, privacy

## Abstract

**Background:**

The environment shapes health behaviors and outcomes. Studies exploring this influence have been limited to research groups with the geographic information systems expertise required to develop built and social environment measures (eg, groups that include a researcher with geographic information system expertise).

**Objective:**

The goal of this study was to develop an open-source, user-friendly, and privacy-preserving tool for conveniently linking built, social, and natural environmental variables to study participant addresses.

**Methods:**

We built the automatic context measurement tool (ACMT). The ACMT comprises two components: (1) a geocoder, which identifies a latitude and longitude given an address (currently limited to the United States), and (2) a context measure assembler, which computes measures from publicly available data sources linked to a latitude and longitude. ACMT users access both of these components using an RStudio/RShiny-based web interface that is hosted within a Docker container, which runs on a local computer and keeps user data stored in local to protect sensitive data. We illustrate ACMT with 2 use cases: one comparing population density patterns within several major US cities, and one identifying correlates of cannabis licensure status in Washington State.

**Results:**

In the population density analysis, we created a line plot showing the population density (x-axis) in relation to distance from the center of the city (y-axis, using city hall location as a proxy) for Seattle, Los Angeles, Chicago, New York City, Nashville, Houston, and Boston with the distances being 1000, 2000, 3000, 4000, and 5000 m. We found the population density tended to decrease as distance from city hall increased except for Nashville and Houston, 2 cities that are notably more sprawling than the others. New York City had a significantly higher population density than the others. We also observed that Los Angeles and Seattle had similarly low population densities within up to 2500 m of City Hall. In the cannabis licensure status analysis, we gathered neighborhood measures such as age, sex, commute time, and education. We found the strongest predictive characteristic of cannabis license approval to be the count of female children aged 5 to 9 years and the proportion of females aged 62 to 64 years who were not in the labor force. However, after accounting for Bonferroni error correction, none of the measures were significantly associated with cannabis retail license approval status.

**Conclusions:**

The ACMT can be used to compile environmental measures to study the influence of environmental context on population health. The portable and flexible nature of ACMT makes it optimal for neighborhood study research seeking to attribute environmental data to specific locations within the United States.

## Introduction

The environment shapes health behaviors and outcomes [1]. Socioecological frameworks that explain behaviors and outcomes as diverse as physical activity [2], bullying [3], violence [4], sexually transmitted infection [5], alcohol-attributable emergency department visits [6], COVID-19 vaccination [7], and sleep [8], posit that the environment in which a person lives and works affects health directly, affects health-related behavior, and may affect the ability to modify health-related behavior successfully [9].

Yet, despite the robust theoretical understanding that built and social environments impact health, literature on environmental influences on health and health behavior remains inconsistent [10]. This is in part because the early evidence base for built environment associations with health has been opportunistic and cross-sectional [10], but it is also because the geographic information systems (GIS) expertise required to develop built and social environment measures at spatial and temporal scales to allow optimal comparison has limited this research to specialist research groups (eg, groups that include a researcher with GIS expertise) [11]. Broadening the research base, not only by exploring environmental exposure hypotheses posited by research groups without specific GIS expertise, but also by studying environmental exposures over time (eg, as in [12] and [13]), and by pooling deidentified data using contextual measures (eg, as in [14]) requires teams to simply and efficiently link environmental measures to existing data.

To fill this gap, we developed a software tool that researchers and practitioners can use to efficiently compile environmental measures drawn from free and nationally available data sets such as the American Community Survey (ACS) [15] and the National Land Cover Database [16], and to spatially link those measures to address data. More importantly, our tool uses a locally installed geocoder and does not share addresses with third parties such as Google Maps [17], thus protecting the sensitive information of the research participants. This tool, the automatic context measurement tool (ACMT), has been used in a prior study [18] and is detailed below. We describe the components that comprise the ACMT, demonstrate 2 use cases for the ACMT, and conclude with a discussion of the ACMT’s utility for research.

## Methods

### Installation and Architecture

The ACMT and its dependencies are available on GitHub [19]. The ACMT is packaged to be run using Docker [20], a technology that lets developers package and run self-contained operating system instances within another computing environment.

The ACMT comprises two components: (1) a geocoder, which identifies a latitude and longitude given a US street address and (2) a context measure assembler, which computes measures from publicly available data sources linked to a latitude and longitude. ACMT users access both of these components using an RStudio/RShiny-based web interface that is hosted within the Docker container [21,22].

**Figure 1:**
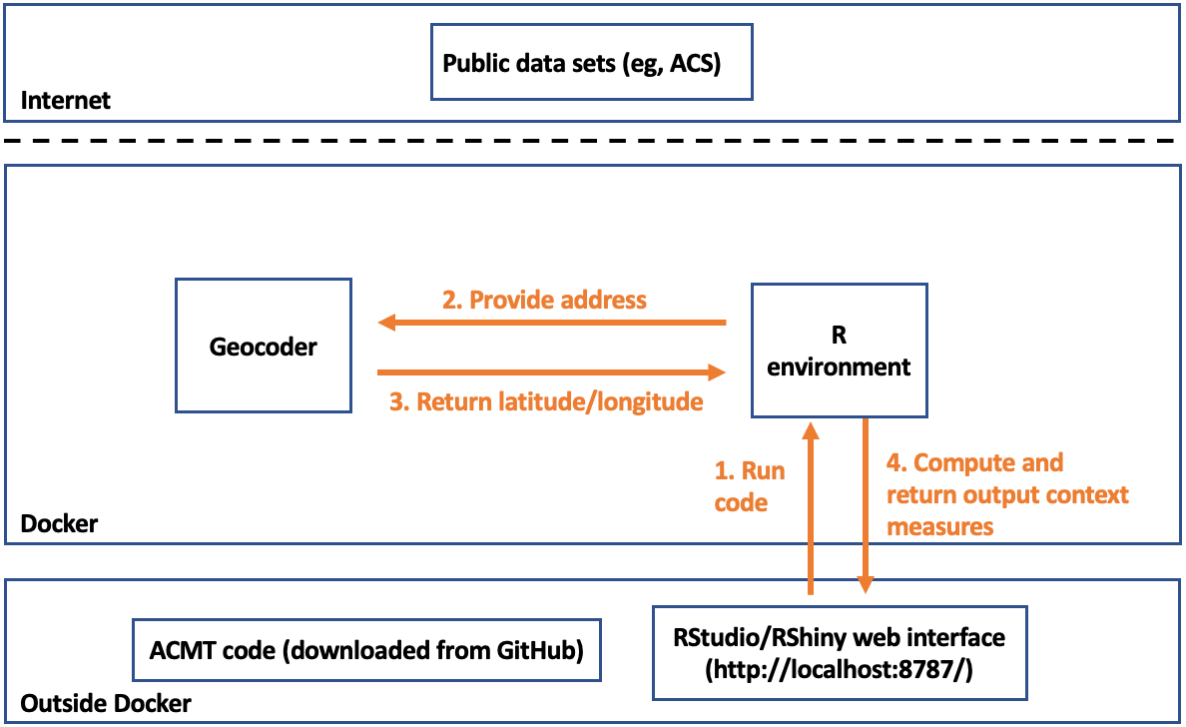
The automatic context measurement tool (ACMT) architecture. The bottom box shows the ACMT code downloaded at the user’s computer and the RStudio/RShiny web interface open at a local browser. The middle box shows the geocoding and R computing happening within Docker. The top box shows the internet layer where ACMT downloads public data sets (eg, ACS) data from but ACMT would not upload sensitive information to the internet. The geocoding and context measure computing only happens within the docker container which is on the user’s local computer. In a typical workflow, from the RStudio/RShiny web interface, a user sends R codes to Docker. Docker then geocodes addresses, assembles the context measures, and sends outputs back to the user. ACS: American Community Survey.

Figure 1 below shows the flow of data in a typical use of the ACMT. First, a data analyst points their browser to the web interface (accessing the Docker container on their local machine). The R environment [23] will be running in the web interface using RStudio/RShiny, depending on analyst preference. That R environment hosts logic that can convert an address (limited to the United States) to its corresponding latitude and longitude (the geocoder) and can download public data from the internet to compile environmental context measures for latitude and longitude (the context measure assembler). The address conversion is performed using the locally installed geocoder, thus preserving privacy. The analyst can then work with these context measures directly in the RStudio interface or can export them for use with other statistical software. The components are described in more detail below.

### Geocoder

A geocoder is a piece of software that, given a street address, identifies the latitude and longitude corresponding to the location identified by the street address. For example, an ACMT user might write code to geocode a location (ie, get latitude and longitude coordinates for the location) such as “600 4th Ave, Seattle, WA 98104, United States” (Seattle City Hall), and the ACMT’s geocoder would supply (47.60, –122.33) as the geographic coordinates of the location corresponding to that address, as well as a rating—an indicator of how confident the geocoder is that the geographic coordinates accurately reflect the location of the street address. The rating ranges from 0 (very high confidence) to 100 (very low confidence). For addresses with lower confidence, users might consider restructuring the address or dropping them.

The ACMT’s geocoder is built off of an open-source PostGIS geocoder [24] and does not share addresses with third parties (ie, unlike Google Maps [17]). This geocoder incorporates street data from TIGER/Line files compiled by the United States Census, which provides the mapping from street names and addresses into latitude and longitude files [25].

In practice, ACMT usually processes thousands of addresses at a time. In some cases where most of the addresses appear to have a very low confidence, it may be an issue with the installed geocoder database and it might be worth checking whether the geocoder database is installed correctly. In cases where only a few of them appear to have very low confidence, we recommend selecting those IDs in the verification checker to do a manual review.

**Figure 2:**
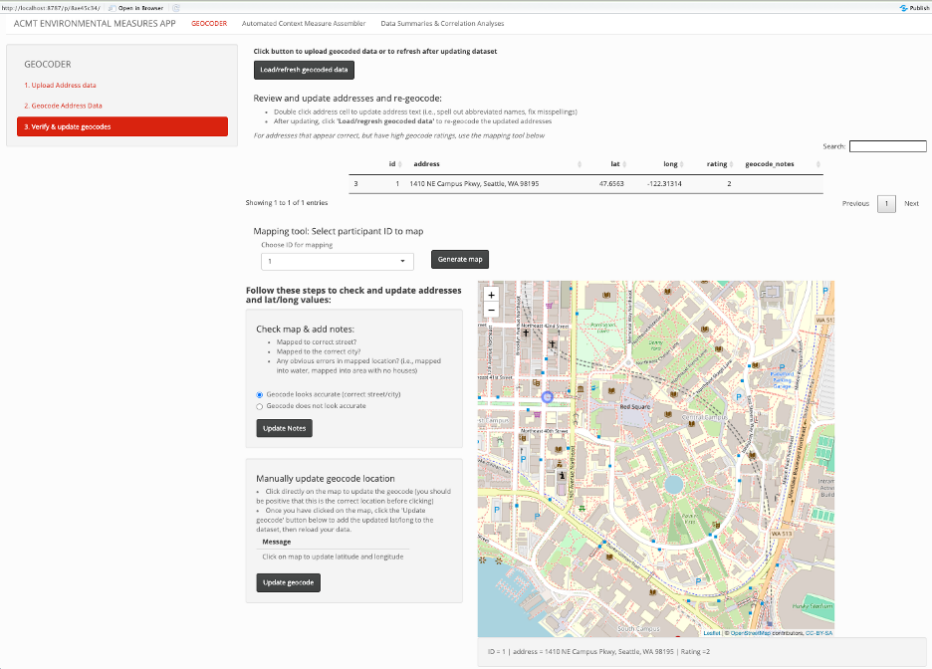
The geocoding verification checker from ACMT’s RShiny interface (here checking the address for “1410 NE Campus Parkway”, the address of the University of Washington). ACMT: automatic context measurement tool.

The ACMT includes a geocoding verification component that, given an address and its latitude and longitude, displays a street map of the location including a pin highlighting the geocoded location that an analyst can use to ensure the geocoder appears accurate (Figure 2). The verification tool also allows the analyst to directly edit addresses to improve geocoding accuracy by writing out abbreviated street names or fixing obvious address errors. Addresses are regeocoded and the updated rating is shown in the verification tool. Analysts can also manually update addresses on the map if they are confident of the exact location of the address and can see that the generated geocodes are incorrect. Importantly, this geocoder verification process never sends the geocoded address over the open internet. By using Leaflet [26], an open-source mapping tool hosted in the local R environment, the host computer downloads map tiles for a general region, annotates the location locally, and does not reveal a specific address. If one wishes to batch process the verification checker, we recommend layman users to contact our support team, and users with R programming experience to consider reusing the provided source code to generate and save images for each ID and view them offline.

### Context Measure Assembler

#### Overview

The context measure assembler takes a location and a neighborhood radius around that location and produces standardized context measures from publicly available data regarding that location. Some common publicly available data include measures from the ACS [15] and administrative data for particular localities. Users can also compile their customized data sets and use them within ACMT. As detailed below, the ACMT supports assembling both areal and point-aggregate context measures.

Currently, the ACMT only supports gathering context measures for a circular area parameterized by a coordinate and a radius. However, we do understand that users might want to gather context measures for an administrative unit such as a census tract or a block group, for example, the number of 911 calls in the Census Tract 82 of Seattle in 2020. We leave this for future work.

#### Areal Context Measures

Areal context measures are defined by aggregating summary statistics that apply to predefined areas. For example, ACS [15] measures are defined in reference to a census unit such as a tract or block groups (eg, count of adults living in the Washington State King County tract).

The ACMT areal context measure assembler identifies the census tract or tracts corresponding to the area defined by a location and neighborhood radius (specified by the user), and then uses area-weighted interpolation to compute a single average measure for that region. For example, if passed 47.60 as the latitude, –122.33 as the longitude for the location (Seattle City Hall), and 1000 m as the neighborhood radius, the assembler would first identify an area defined by a 1000 m circle centered at (47.60, –122.33), then compute measures reflective of that area. These measures might include the estimated count of adults living in this area, the average proportion of that adult population who had completed high school, or the average proportion of employed adults living in that area who reported commuting by bicycle, etc.

**Figure 3:**
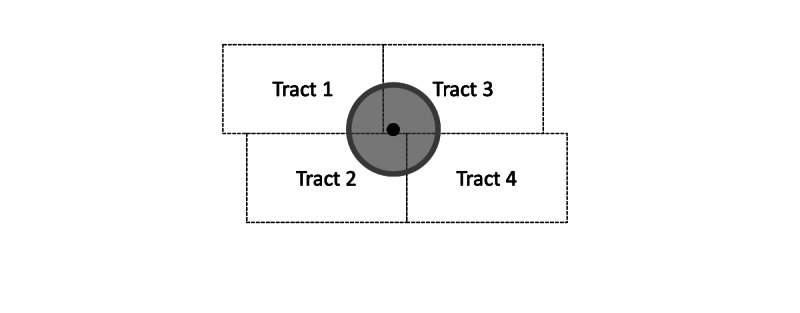
Illustration of area-weighted interpolation. Suppose the black dot represents the location of interest (address) and the gray circle represents the neighborhood (eg, 1000 m surrounding the location of interest). Then an area-weighted interpolation represents the sum of the estimate for tract 1 weighted by the proportion of tract 1 falling in the gray circle, plus the estimate for tract 2 weighted by the proportion of tract 2 falling in the gray circle, plus the estimate for tract 3 weighted by the proportion of tract 3 falling in the gray circle, and plus the estimate for tract 4 weighted by the proportion of tract 4 falling in the gray circle.

The area-weighted interpolation that converts area tract estimates to radial buffer estimates follows a conventional area-weighting approach (Figure 3). First, given the location coordinates (ie, latitude and longitude) and the radius, the assembler computes a buffer corresponding to a circle centered on the location with the given neighborhood radius, accounting for the earth’s curvature by projecting on state plane projections as defined by the National Oceanic and Atmospheric Administration’s National Geodetic Survey [27]. Next, using a spatial overlay, the assembler identifies the census tracts (or other geographical units depending on the data set) that intersect that radial buffer and partition the radial buffer into pieces wherein each piece falls into exactly one census tract. The assembler then computes the context measure for each piece by assuming the tract-specific measure is uniform across the tract, and thus the measure for the piece of the context buffer is proportional to the overall measure for the tract. Finally, to compute a measure for the whole buffer, the assembler combines the pieces of context measures back to one either by summing or area-weighted averaging, depending on the type of measure. The context measures that are combined by summing are count-based measures, such as population and the amount of open space; the context measures that are combined by averaging are proportion-based measures, such as the proportion of the population with high school diplomas and the proportion of the population who commute by bike.

To illustrate, suppose the ACMT was used to estimate the population in the gray area shown in Figure 3. Suppose the circle intersects 6% area of census tract 1, 12% of census tract 2, 12% of census tract 3, and 6% of census tract 4, and suppose tracts 1, 2, 3, and 4 have a population of 2000, 18,000, 3000, and 1800 respectively. The population estimate assembled for the gray circle would then be 6% × 2000 + 12% × 1800 + 12% × 3000 + 6% × 1800 = 804.

Currently, ACMT assembler is weighted by the area of the tracts. We understand users might want to have other measures for weighting the tracts such as the population density, and we leave them as future work.

#### Point-Aggregate Context Measures

Whereas areal context measures aggregate data representing areas, point aggregate context measures assemble areal measures from data recorded at points, wherein each point has its own corresponding latitude and longitude. For example, addresses from which 911 calls originated and the distribution of Airbnb [28] short-term rentals in the United States are point measures.

**Figure 4:**
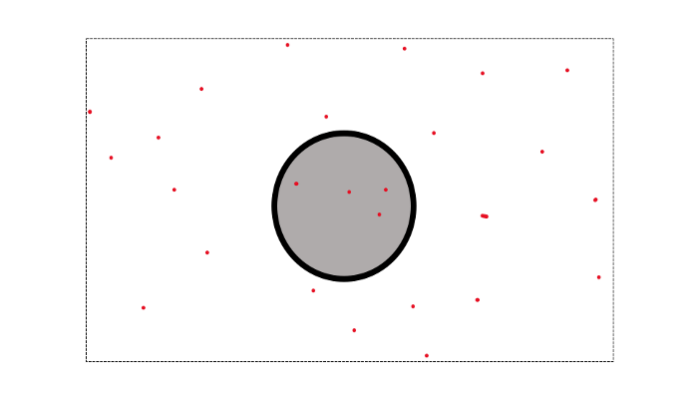
Point-aggregate interpolation. Suppose the black dot represents the location of interest, the gray dot represents the neighborhood, and the red dots indicate 911 calls. A point-aggregate sum would indicate that four 911 calls fell within the neighborhood area.

In assembling such measures, ACMT uses point-interpolation to estimate the number of instances that fall within the radial buffer. As an example, a common use of ACMT is to estimate the number of 911 calls in an area. In Figure 4, the red dots are the location of 911 calls by longitude and latitude, and we want to estimate the number of 911 calls that occur within the radial buffer. ACMT will overlay the radial buffer on the coordinates and estimate the number of 911 calls that fall into it. In this example, four 911 calls happened within the specified area.

### Data Sets

Many data sets are publicly available and spatially precise. The ACMT has built-in code to make common data sources available (Table 1), and also provides easily modified code templates for custom data sets.

**Table 1:** Data sets that have been successfully integrated for use with the automatic context measurement tool (ACMT).

Data set	Description	Context measure type	Geographical unit
American Community Survey (ACS) [15]	American Community Survey measures of the United States Census Bureau	Areal	Census Tract
Modified Retail Food Environment Index (mRFEI) [29]	Health-related index as measured by the number of healthy and less healthy food retailers in the United States	Areal	Census Tract
Smart Location Database Walkability Index [30]	The walkability scores in the United States	Areal	Block Group
National Land Cover Database (NLCD) [16]	The land cover statistics of the United States	Areal	30 m Grid across the US
Seattle 911 Call [31]	911 calls in Seattle	Point-aggregate	Coordinate
Seattle Crime [32]	Crime incidents in Seattle	Point-aggregate	Coordinate

### ACMT User Interface

**Figure 5:**
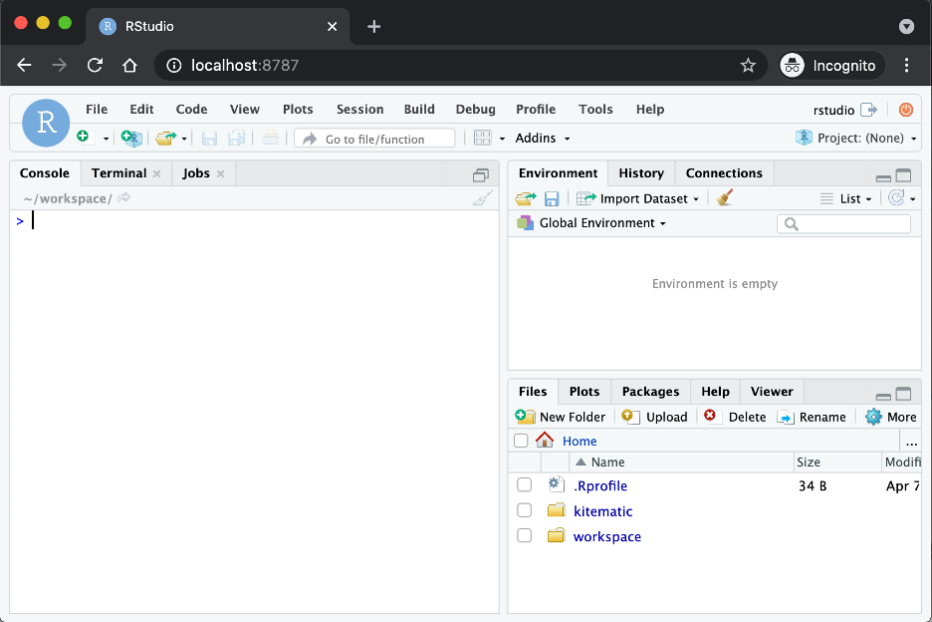
Automatic context measurement tool (ACMT) RStudio user interface. An analyst can use conventional RStudio features, including the editor and debugger, to access the ACMT geocoder and the context measure assembler.

The ACMT has 2 levels of user interface, adjusting for users with different backgrounds. The first, which is the most flexible, offers access to ACMT features via RStudio hosted within the data analyst’s locally installed Docker instance (Figure 5). Using the RStudio interface, a data analyst can write R code that uses ACMT functions for geocoding addresses and request environmental measures. With this interface, a user can upload environmental data sets to interpolate for their geocoded data sets, use data sets publicly available on the internet, or use one of the environmental measures data sets that are already set up in the ACMT functions (ACS, Modified Food Retail Environment Index, National Land Cover Database, CDC Places, Walkability Index, etc).

**Figure 6:**
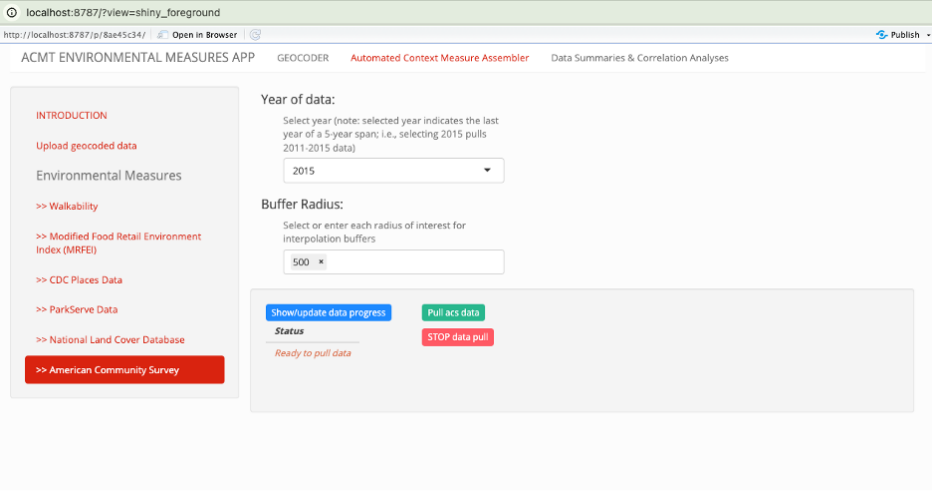
ACMT RShiny user interface for analysis using the American Community Survey data set. An analyst can select parameters visually for performing analysis. ACMT: automatic context measurement tool.

The second interface, which is more friendly to layman users and avoids requiring the data analyst to write R code, uses RShiny-based helper screens to guide analysts through a process by which they open a file of locations (eg, street addresses), geocode them, specify a data set, a neighborhood radius and years of data to spatially link, allowing the ACMT to perform the appropriate data download, spatial linkage, and interpolation for the analyst’s data set of interest. Currently, the RShiny interface is limited to these publically available data sets: ACS, Walkability, Modified Retail Food Environment Index, National Land Cover Database, etc. Figure 6 shows the RShiny interface for linking to ACS data.

### Worked Examples of ACMT Use

#### Population Density in Relation to Distance From City Hall

Suppose a researcher is interested in understanding the spatial scale at which residential population density drops off as distance increases from city hall: the shape of such a curve might be a rough indicator of how much a metropolitan area is affected by urban sprawl, wherein less sprawling metro areas might see population density drop off more quickly. The ACMT would be suited to this task.

Specifically, we select Seattle, Los Angeles, Chicago, New York City, Nashville, Houston, and Boston to represent large metropolitan areas with different histories and different geographic constraints on urban growth. We first use the ACMT geocoder to geocode the addresses of the respective city halls in these central cities. We then select 1000, 2000, 3000, 4000, and 5000 m as radial distances to compare. Using the ACMT, we compute the population per square meter of land area (ie, to eliminate water) of each region within each radial distance, plot the density-to-distance-from-city-hall curve for each city, and compare. The detailed steps are included in ACMT as an example.

#### Neighborhood Environment-Wide Association Study to Assess Correlates of Cannabis Retail License Status

A second use case for the ACMT might be to compile measures for a Neighborhood Environment-Wide Association Study (NE-WAS), a study that assesses the strength of association between neighborhood measures and some outcome of interest [18,33,34]. While NE-WAS studies often focus on residential environments, we provide an example NE-WAS using public addresses so that the code for the example could be shared as part of the ACMT code download. An analyst can download the publicly available Seattle dispensary address data and run the full example for reference. In this example, we assess the characteristics of neighborhoods around Washington State cannabis retail license applicants and their association with license approval status.

### Ethical Considerations

This study does not involve human participants and it does not require institutional review board approval. All the data used in this study were collected by the third-party and the data sets were publicly released. This study also used deidentified data only. No identifiable information of individual participants/users was included in this manuscript.

## Results

### Population Density in Relation to Distance From City Hall

**Figure 7:**
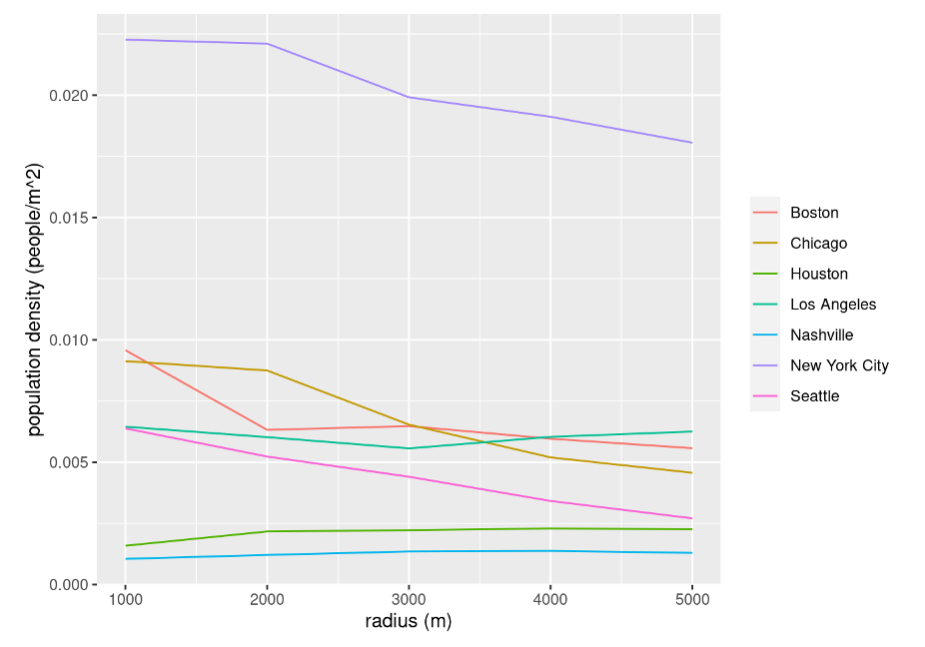
The population density in relation to distance from the city hall for Seattle, Los Angeles, Chicago, New York City, Nashville, Houston, and Boston with the distances being 1000, 2000, 3000, 4000, and 5000 meters.

As shown in Figure 7, population density per land area across tended to decrease as distance from city hall increased except for Nashville and Houston, 2 cities that are notably more sprawling than the others. The population density of Nashville is around 0.001 across all distances and the population density of Houston is around 0.002 across all distances.

Other cities all have population densities above 0.002 for all distances. The population density of Seattle is around 0.006 at 1000 m away from the city hall, and it drops to around 0.002 at 5000 m away from the city hall, dropping by more than a half. In comparison, the population density of Los Angeles is also around 0.006 at 1000 m away from the city hall, and it remains similar at 5000 m away from the city hall. This could be because Los Angeles is a larger metropolitan area than Seattle and its population is more evenly spread throughout its urban core. Besides, Seattle is a city surrounded by water and this geographical characteristic makes it difficult to expand efficiently, while Los Angeles is a land-based city and this geographical characteristic makes it easier to expand.

Chicago and Boston both have a similar population density of around 0.01 at 1000 m from city hall, but they experience different trends when getting further from the city hall. Chicago has a steady drop in population density from around 0.01 to around 0.005. Boston has a quick population drop at 2000 m, dropping from 0.01 to 0.005, but remains relatively stable afterward. This could be because the Boston City Hall is located at a business center on a peninsula that has a radius of around 2000 m, so the population drops suddenly when the population density calculation starts to cover lands outside the peninsula. The Chicago City Hall is near Lake Michigan. It has a land area on the west side and a water area on the east side, thus resulting in a relatively stable population density when we increase the distance from 1000 m to 5000 m.

As expected, New York City has a significantly higher population density than all the other cities, with a population density of around 0.023 at 1000 m and a population density of around 0.017 at 5000 m. The population density remains steady at 1000 m and 2000 m away from the city hall, but it has a sudden drop at the distance of 3000 m, and this could be because this distance is reaching outside of Manhattan and starting to cover the less dense areas such as Jersey City and Brooklyn.

### NE-WAS to Assess Correlates of Cannabis Retail License Status

**Figure 8:**
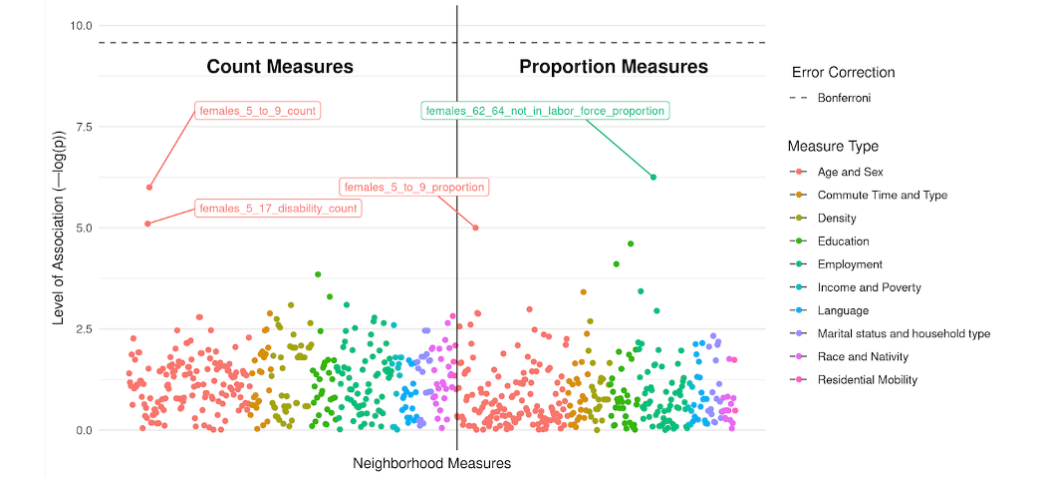
Manhattan plot of regression coefficients for the association between neighborhood characteristics and cannabis license status (eg, active and open vs closed). Each dot represents one environment measure, and the y-axis represents the strength of association. Measures are grouped by the domain of environment measured and by whether the measure was a count (eg, number of residents within 1000 m of an outlet who were females aged 5 to 9 years) or proportion (eg, percentage of residents within 1 km of an outlet who were females aged 5 to 9 years).

In our NE-WAS study (Figure 8), a few environmental measures have a level of association greater than the others. For the count measures, the 2 measures that have the highest level of association are females aged 5 to 9 years, with a level of association of around 6, and females aged 5 to 7 years with disability, with a level of association of around 5. For the proportion measures, the 2 measures that have the highest level of association are females aged 62 to 64 years who are not in the labor force, with a level of association of around 6, and females aged 5 to 9 years, with a level of association of around 6. We observed that females aged 5 to 9 years are both among the highest associated count or proportion measures. The strongest predictive characteristic of cannabis license approval is the count of female children aged 5 to 9 years and the proportion of females aged 62 to 64 years who are not in the labor force. Regulations for dispensaries in Seattle require licensed retailers to be at least 1000 feet from schools and playgrounds and at least 500 feet from child-care centers, recreation centers, libraries, parks, or transit centers [31], which may explain the association that we see between females aged 5 to 9 years and females aged 5 to 17 years with disabilities and licensure approval. Nevertheless, after accounting for Bonferroni error correction, none of the measures were found to be significantly associated with cannabis retail license approval status.

## Discussion

### Principal Findings

We developed the ACMT, a tool that compiles environmental measures to study the influence of environmental context on population health. We then demonstrated its use to explore population density in relation to distance to town hall and environmental correlates of cannabis retail license status. These examples, for which code is available in the online supplement, illustrate how the ACMT can be used to construct geospatial data sets without sending identifying information on the open internet. The ACMT is available for download at [19].

The ACMT is distinct from other neighborhood measurement approaches for studies of health and place. Whereas GIS like Arc Geographic Information System [35] and Quantum Geographic Information System are general-purpose tools for geospatial data management and analysis, the ACMT is a streamlined system designed for the specific purpose of computing environmental measures specific to a location (longitude and latitude). Yet unlike other low-cost alternatives such as entering subject addresses into Google Maps [17], the ACMT approach preserves participant privacy and compliance with typical institutional review board concerns by ensuring this measure computation happens fully within the analyst’s local computer [32,33,36,37].

The ACMT has several key strengths. It is free for use, with a team of researchers dedicated to making it serve the research needs of potential users. It allows for a simple, privacy-preserving assembly of measures from public data sets, unlocking the potential to better understand place-based disparities in health and health behavior. Additionally, the user-friendly RStudio and RShiny interfaces allow for simple paste-code/edit-code or point-and-click interactions that do not require analysts to parse complicated GIS concepts like data layers or projections.

However, the ACMT also comes with limitations. The Docker-based packaging system, though necessary to ensure access to a geocoder while also ensuring study participant data does not leave the local machine, can be an installation challenge in tightly locked-down environments. For example, we found in early deployments of the ACMT that some information technology systems in some academic medical center settings set firewalls to preclude the Docker instance from running a webserver for local access. While these issues can be worked around with IT support, they are nonetheless a challenge. Furthermore, as the ACMT is not a general-purpose GIS system, complex geospatial operations including alternate interpolation strategies or road network buffers are not supported at this time. Finally, the ACMT’s utility is intrinsically tied to the quality of available data—if a researcher’s environmental constructs are not well captured by public data, the ACMT cannot help assess it.

### Conclusion

ACMT is a user-friendly piece of software that runs in an isolated environment and can be easily installed for Windows, MacOS, and Linux. Users with no R coding experience can use the RShiny interface to pull commonly used measures, while more advanced R users can use the ACMT code package and an RStudio (Posit PBC) interface to pull from any available environmental data sets. Both approaches avoid sending personally identifying information to third parties. The ACMT thus unlocks access to contextual measures for researchers without geographic information systems training, which in turn both enables individual research teams to explore context in relation to health outcomes and allows for larger consortia to pool data sets with contextual information while preserving privacy.

## Abbreviations

ACMT: automatic context measurement tool
ACS: American Community Survey
GIS: geographic information systems
NE-WAS: Neighborhood Environment-Wide Association Study

## Data Availability

The data sets analyzed during this study and the automatic context measurement tool (ACMT) software are available in the ACMT-network repository [19].
